# Mitochondrial Factor C20orf7 Facilitates the EMT-Mediated Cancer Cell Migration and the Proliferation of Colon Cancer In Vitro and In Vivo

**DOI:** 10.3390/genes13112111

**Published:** 2022-11-14

**Authors:** Hou-Hsien Liu, Chia-Hwa Lee, Yi-Chen Hsieh, Jia-Huei Zheng, Yun-Ru Liu, Chia-Hsuan Chang, Er-Chieh Cho

**Affiliations:** 1 School of Pharmacy, College of Pharmacy, Taipei Medical University, Taipei 110, Taiwan; 2 School of Medical Laboratory Science and Biotechnology, College of Medical Science and Technology, Taipei Medical University, Taipei 110, Taiwan; 3 Ph.D. Program in Medical Neuroscience, College of Medical Science and Technology, Taipei Medical University, Taipei 110, Taiwan; 4 Ph.D. Program in Biotechnology Research and Development, College of Pharmacy, Taipei Medical University, Taipei 110, Taiwan; 5 Joint Biobank, Office of Human Research, Taipei Medical University, Taipei 110, Taiwan; 6 Master Program in Clinical Genomics and Proteomics, College of Pharmacy, Taipei Medical University, Taipei 110, Taiwan; 7 Cancer Center, Wan Fang Hospital, Taipei Medical University, Taipei 110, Taiwan; 8 TMU Research Center of Cancer Translational Medicine, Taipei Medical University, Taipei 110, Taiwan

**Keywords:** mitochondrial factor, C20orf7, epithelial–mesenchymal transition (EMT), 5-fluorouracil (5FU), colon cancer progression, therapeutic target

## Abstract

Colon cancer is a major malignant neoplasm with a low survival rate for late-stage patients. Therefore, the investigation of molecules regulating colon cancer progression and the discovery of novel therapeutic targets is critical. Mitochondria play a vital role in maintaining the homeostasis of cells. Abnormal mitochondrial metabolism alterations and the induction of glycolysis can facilitate tumor growth; therefore, targeting mitochondrial molecules is suggested to be a promising strategy for cancer treatment. In this study, we investigated the role of this largely unknown mitochondrial factor, chromosome 20 open reading frame 7 (C20orf7), in colon cancer progression. Clustered regularly interspaced short palindromic repeats (CRISPR) technology was utilized for C20orf7 depletion, and functional assays were performed to examine the regulation of C20orf7 in colon cancer cells. We demonstrated that C20orf7 facilitates epithelial–mesenchymal transition (EMT)-mediated cell migration and promotes the proliferation of colon cancer. The anti-cancer drug 5-fluorouracil (5FU) was also applied, and C20orf7 was targeted with a combination of 5FU treatment, which could further enhance the anti-cancer effect in the colon cancer cell line and the xenograft mice model. In summary, this study demonstrated, for the first time, that C20orf7 plays a promotional role in cancer tumorigenesis and could be a promising therapeutic target in colon cancer treatment.

## 1. Introduction

Colon cancer has long been a major malignant neoplasm worldwide, especially in developed countries [1]. The incidence and mortality of colon cancer rank third in men and second in women among all types of cancer globally [2]. Factors include unhealthy diet habits, obesity, smoking, lack of exercise, family history, and inflammatory bowel diseases, etc., which are all common causes of the disease [3,4,5,6,7]. Cancer treatments include surgery, chemotherapy, targeted therapy and radiation therapy, and molecular biomarkers play critical roles in the therapies [8,9]. Unfortunately, the survival rate of late-stage patients is low [10]. The five-year survival rate of stage I patients is 93.2%, but this can drop to 8.1% when cancer progresses to stage IV according to the statistical data from the American Joint Committee on Cancer (AJCC).

The major function of mitochondria is to generate energy for cells by providing ATP and metabolites. The process of energy production, called oxidative phosphorylation, is mediated by electron transfer between large protein complexes I to V within the inner mitochondrial membrane area [11]. Although mitochondria exist in most eukaryotic cells, the number, size, and metabolism of mitochondria in different cell types and cells in different cell-cycle stages can vastly differ [12]. Intact mitochondria capacity is crucial for maintaining the homeostasis of cells [12]. It was suggested that tumor cells heavily utilize glycolysis and mitochondrial metabolism to acquire sufficient nutrients and ATP to promote proliferation in cells [13]. The inactivation of tumor suppressors or activation of oncogenes can also result in mitochondrial metabolism alterations and glycolysis induction, thereby facilitating tumor growth [14,15,16]. Therefore, targeting molecules within the mitochondria or the ATP production pathway is suggested as a promising strategy for cancer treatment [12,17].

Chromosome 20 open reading frame 7 (C20orf7), also known as NADH:ubiquinone oxidoreductase complex assembly factor 5 (NDUFAF5), is a mitochondrial respiratory factor that is important for complex I assembly at an early stage [18,19,20]. The biochemical function of C20orf7 is largely unclear; however, it was suggested that to be a member of the S-Adenosyl methionine (SAM)-dependent methyltransferase family, and the mutation of C20orf7 leads to complex I defection and could result in the neurological disorder Leigh syndrome [18,21]. Although one study reported that C20orf7 was overexpressed in lung cancer, and another found that C20orf7 was downregulated with the depletion of oncogenic MRG-binding protein by siRNA in colon cancer cells [22,23], our knowledge of C20orf7 is minimal.

In our group, C20orf7 was identified to be upregulated when we previously analyzed samples of DLD-1 colon cancer cells with an overexpressed oncogene. In this study, we further investigated the role of C20orf7 in colon cancer regulation.

## 2. Materials and Methods

### 2.1. Cell Culture, Stable Cell Line Establishment, and Plasmids

Human colon cancer cells, DLD-1 (American Type Culture Collection), were cultured in RPMI medium (Gibco, Waltham, MA, USA) with 5–10% fetal bovine serum (FBS) and 1% antibiotic-antimycotic (Gibco, USA) in a humidified incubator with 5% CO_2_ at 37 °C. Stable cell lines were established by infection with lentivirus for C20orf7 gene knockout. The virus contained clustered regularly interspaced short palindromic repeats (CRISPR) and associated plasmids that can facilitate downregulation of C20orf7. Established stable cell lines were scramble control, namely C-Scr, and the one with knockout C20orf7 gene, namely C-C20orf7. Cell selection was then carried out by puromycin (Cayman Chemical, Ann Arbor, MI, USA). Overexpressing plasmids were from Origene.

### 2.2. Construction of CRISPR/Cas9 Plasmid and Production of Lentivirus

Lentivirus was generated as described previously [24] with transient transfection (TransIT-LT1 Reagent, Mirus Bio LLC, Madison, WI, USA) and Phoenix-ECO cells (CRL-3214). Phosphorylated guide oligonucleotides were cloned into the lentiCRISPR v2 vector plasmid (Addgene and Dr. Feng Zhang, Watertown, MA, USA) at BsmBI site following the protocol from Zhang laboratory (MIT, Cambridge, MA, USA) and as described [25]. Constructed plasmids were all analyzed and confirmed by sequencing. The lentiCRISPR construct and pMD2.G (Addgene #12259) and psPAX2 (Addgene #12260) (both from Dr. Didier Trono, Switzerland) were co-transfected into the cells. At 36 h and 72 h time points, viral particles were collected. Lenti-X Concentrator (Clontech, Mountain View, CA, USA) was used for concentrating the viral particles.

### 2.3. Sanger Sequencing Analysis and Gene Editing Evaluation

Extraction of genomic DNA from CRISPR stable cells and PCR amplification of C20orf7 exon 1 region were performed as described before [24]. PCR primers are listed here: forward 5′-GCATGCGCACAAAAAGC-3′ and reverse 5′-TGCAATGTAACCTCTTCCAC-3′. PCR product was purified and then sequenced with the PCR forward primer by Sanger sequencing. The TIDE software (Netherlands Cancer Institute; https://tide.nki.nl/ (accessed on 19 April 2018)) was applied to evaluate the sgRNAs editing efficiency and also the potential mutations.

### 2.4. Transfection

Overexpressing plasmids used are listed here. EGFP and pcDNA control plasmids were kindly provided by Dr Yun Yen, Taiwan. C20orf7-DDDDK plasmids was from Origene (RG210078). Cells were seeded the night before the transfection. The next day, DNA plasmids and the transfection reagent (T-Pro, New Taipei, Taiwan) were used for transfection. Plasmids, 0.15µg per well for 96-well plates or 2.4µg per well for 6-well plates, were firstly mixed with the transfection reagent in a ratio 1:1 and added into the serum-free medium (RPMI). Reagent was mixed gently. The mixture was then incubated for 15 min at room temperature before being added drop by drop into the cells. After 48 h, cells were harvested for Western blot analysis or retained for other functional assay assessments.

### 2.5. Western Blot Assay

Cells were treated and then harvested for cell lysis by mixing with lysis buffer (Promega, Madison, WI, USA) containing protease inhibitor (Roche) and phosphatase inhibitor (MedChem Express, Princeton, NJ, USA). Samples were vortexed and then kept at −80 °C overnight, then centrifuged at 13000 rpm, 4 °C for 5 min. Supernatant was collected for concentration measurement by protein assay reagent (Thermo Fisher Scientific, Waltham, MA, USA). A 40 µg sample of proteins was loaded in each lane for SDS-PAGE analysis. Next, proteins were transferred from gel to polyvinylidene difluoride membrane (PVDF, Pall, New York, NY, USA). A 5% measure of skim milk in Tris-buffered saline with 0.1% tween 20 (TBST) was used for blocking. After the blocking process, membranes were incubated with primary antibody in TBST at 4 °C on the shaker overnight, washed with TBST, and then subjected to secondary antibody for 1 h at room temperature. Finally, membranes were washed and then protein levels were visualized by enhanced chemiluminescence (ECL, Advansta, San Jose, CA, USA) [26]. GAPDH (GeneTex, San Antonio, TX, USA) and C20orf7 (GeneTex, San Antonio, TX, USA) antibodies were applied.

### 2.6. MTS Cell Proliferation Assay

Ninety-six-well culture plates were used for the assay. Three thousand cells were seeded in each well overnight. The next day, cells were treated where applicable, and then harvested at the indicated time points. A 10 µL sample of MTS reagent (Promega, Madison, WI, USA) was added to each well, and then incubated at 37 °C for 1 h. Absorbance at 490 nm wavelength was measured by microplate spectrophotometer (BioTek Instruments, Inc., Winooski, VT, USA) [27]. *N* = 3 for each experiment.

### 2.7. Colony Formation Assay

Six-well culture plates were used for this assay. One hundred cells were seeded in each well with 2 mL of medium, and treatments were applied the next day where applicable. A measure of 0.5 mL complete medium was added to each well every 3 days. After 12 days, medium was removed, and 0.5 mL of 0.5% crystal violet (dissolved in acetic acid/methanol 1:3) agent was added to each well to fix and stain the colonies for 15–20 min. Finally, the plates were air dried for colony number calculation and analysis [28]. *N* = 3 for each experiment.

### 2.8. Wound Healing Assay

Either 6-well or 12-well culture plates were used. Quantities of 1 × 10^6^ cells (for 6-well plates) or 5 × 10^5^ cells (for 12-well plates) were seeded in each well. Cells were treated where applicable; then, scratches were made by 200 µL tips when cells reached 90% density. Cells were monitored and pictures were taken at each indicated time point. The migration rate in each group was calculated at indicated time points. Migration rate is defined as (distance at 0 h—distance at each time point)/distance at 0 h [29]. *N* = 3 for each experiment.

### 2.9. Transwell Migration Assay

Cells were transfected, and then, the next day, mixed with serum-free medium and seeded on the upper layer of the Transwell insert (Millipore, Burlington, MA, USA) at a density of 3 × 10^4^ cells per well, while FBS-containing medium was in the lower part. At 48 h time point, cells on the upper surface were wiped off gently with a cotton swab, whereas those on the lower side were stained with 0.1% crystal violet solution. Images of migrated cells were captured under the microscopy, and cells were calculated for quantification, and the number in the experimental group was compared with that in the control group [28].

### 2.10. Xenograft Experiment

Animal studies were conducted following the guidelines of the Animal Care and Use Committee of Taipei Medical University (protocol code LAC-2019-0555). Six-week-old male nude mice (National Laboratory Animal Center, Taiwan) were applied in this experiment. The mice were quarantined for one to two weeks before starting the experiment. A measure of 1 × 10^7^ DLD-1 stable cells (C-Scr or C-C20orf7) was injected into the flank of the right thigh of the mice subcutaneously to establish the xenograft mice model. A week later, we started the treatment when the size of the tumors reached 1 mm^3^. The 5-fluorouracil (5FU) (5 mg/kg) (Cayman Chemical, Ann Arbor, MI, USA) treatment was administrated and provided through intraperitoneal injection three times a week where applicable. The body weight and the tumor size were measured by digital caliper twice a week, and the tumor volume was calculated in mm^3^ by the formula: V = (L × W × W)/2, where L stands for the length of the tumor and W stands for the width of the tumor [30]. Tumor growth inhibition, tumor volume of the treatment group(T)/tumor volume of the control group(C) %, was calculated. According to the National Cancer Institute (NCI), a T/C% ratio standard of equal to or less than 42% would be recognized as effective tumor inhibition [31]. At the end of the experiment, the animals were sacrificed, and the blood samples were harvested from them and sent for hematologic analysis (Laboratory Animal Center, Taipei Medical University, Taipei, Taiwan). *N* = 5 for each group.

### 2.11. Statistical Analysis

The comparison between each group was performed by Mann–Whitney U-test or Kruskal–Wallis test. A multiple post hoc test was analyzed by a SAS Macro analysis [32]. *p*-value < 0.05 was considered to reach a significant difference. The statistics were performed using SAS software (vers. 9.4; SAS Institute, Cary, NC, USA).

## 3. Results

### 3.1. Development of C20orf7 Depletion Cell Line Using the CRISPR/Cas9 System

**Figure 1:**
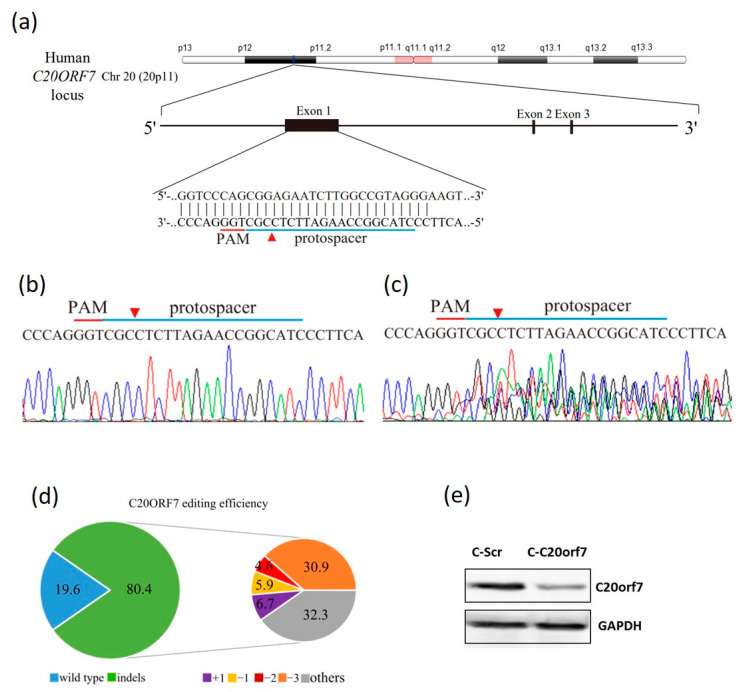
Development of chromosome 20 open reading frame 7 (C20orf7) depletion cell line with the clustered regularly interspaced short palindromic repeats (CRISPR)/Cas9 technology. (**a**) Schematic illustration of C20orf7 DNA locus and the blue underline indicates the edited protospacer sequences. The red arrowhead represents the Cas9 cleavage site, and the red underline points out the protospacer adjacent motif (PAM). Lentivirus delivered the sgRNAs into cancer cells for targeting C20orf7, and then C20orf7 exon 4 was analyzed by Sanger sequencing; (**b**) wild-type C20orf7 sequences in the C-Scr cells; (**c**) delivery of sgRNA resulted in mixed sequences around the targeting site in pooled edited cells (the C-C20orf7 cells); (**d**) TIDE software algorithm analysis showed high C20orf7 gene-editing efficiency in cancer cells in the pie chart (indels, insertions and deletions). The efficiency of gene editing mediated by sgRNA is shown in green. Other mutations (32.3%) and −3-bp insertions (30.9%) were the most frequent mutations within the C20orf7sgRNA cell pool; (**e**) C20orf7 protein expression in C-Scr and C-C20orf7 cells was analyzed by Western blot. GAPDH served as a loading control.

To research the role of C20orf7 in colon cancer regulation, the CRISPR/Cas9 genome editing technique was utilized in DLD-1 cells. The custom-designed protospacer targeted the chromosome 20p.11 of the C20orf7 gene locus, as shown in the schematic map (Figure 1a). The transduction of DLD-1 cells with the control scrambled lentivirus resulted in a wild-type C20orf7 sequence without gene editing (Figure 1b), while the transduction of cells with the C20orf7sgRNA lentivirus produced multiple gene disruptions around the cleavage site (Figure 1c, red arrowhead). Moreover, C20orf7sgRNA delivery led to significant gene-editing efficiency: 80.4% of the cell pool was edited (Figure 1d). The control cell line (C-Scr) and the cell line with C20orf7 gene-edited (C-C20orf7) were established. Western blot assay was applied to examine the C20orf7 protein expression level within these two stable CRISPR cells (Figure 1e).

### 3.2. C20orf7 Promotes the Migration Potential of Colon Cancer Cells

**Figure 2:**
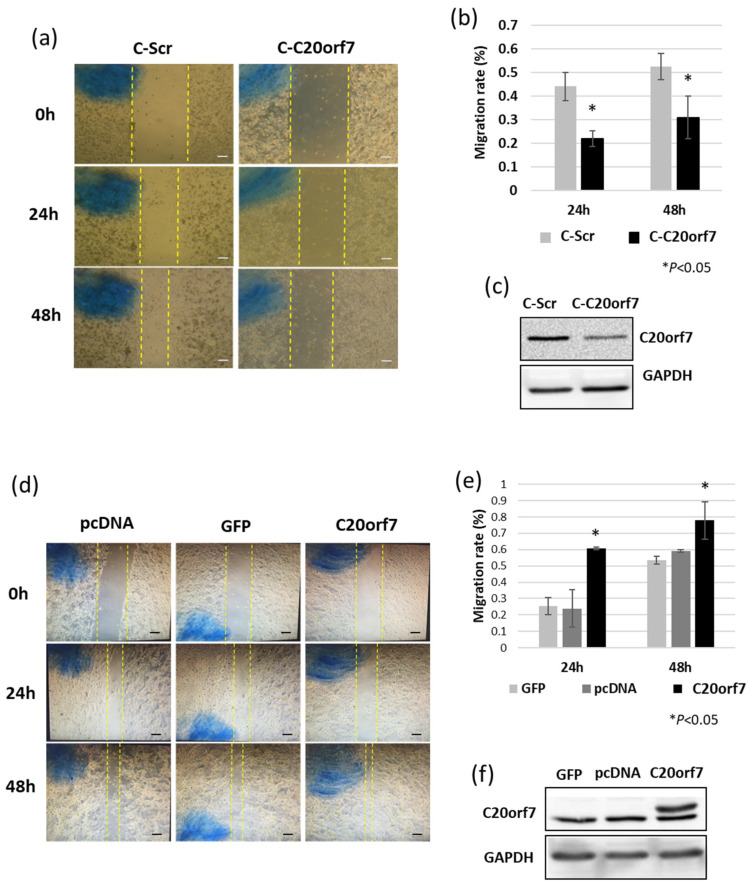
C20orf7 promotes the migration capacity of colon cancer cells. (**a**) Wound healing assay was performed with C-Scr and C-C20orf7 cells. Cells were seeded, scratched, and examined at indicated time points; (**b**) the migration rate of C-Scr and C-C20orf7 cells were analyzed; (**c**) protein expression analysis by Western blot as a control for CRISPR cells. GAPDH was used as control; (**d**) control or C20orf7-overexpressing plasmids were transfected into cancer cells, scratched, and examined at indicated time points under the microscope; (**e**) the migration rates were analyzed in the bar chart; (**f**) protein expression analysis by Western blot assay with control or C20orf7 overexpression samples. GAPDH was used as control. The results are presented as mean ± standard deviation (SD). * *p* < 0.05 was considered as statistically significant. The yellow dashed lines indicate the edges of the cells. The scale bars indicate 100 µm.

The migrating ability of cancer cells characterizes the potential for tumor invasion and metastasis. Therefore, we first investigated whether C20orf7 is involved in cancer migration regulation using a two-dimensional (2D) wound healing assay in CRISPR cells. The results showed that C-C20orf7 cells displayed a slower migration rate compared to C-Scr cells (Figure 2a,b). A representative example of the C20orf7 expression of these cells was analyzed by Western blot assays to confirm the downregulation of C20orf7 in C-C20orf7 cells (Figure 2c). However, control or C20orf7-overexpressing plasmid was transfected in cells and also analyzed by this assay. C20orf7-overexpressing cells migrated faster than controls (Figure 2d,e). A representative example of C20orf7 overexpression was analyzed by Western blot assays (Figure 2f). These results suggested that C20orf7 promotes the 2D migration potential of colon cancer cells.

### 3.3. C20orf7 Promotes the Epithelial–Mesenchymal Transition (EMT) Effect of Colon Cancer Cells

**Figure 3:**
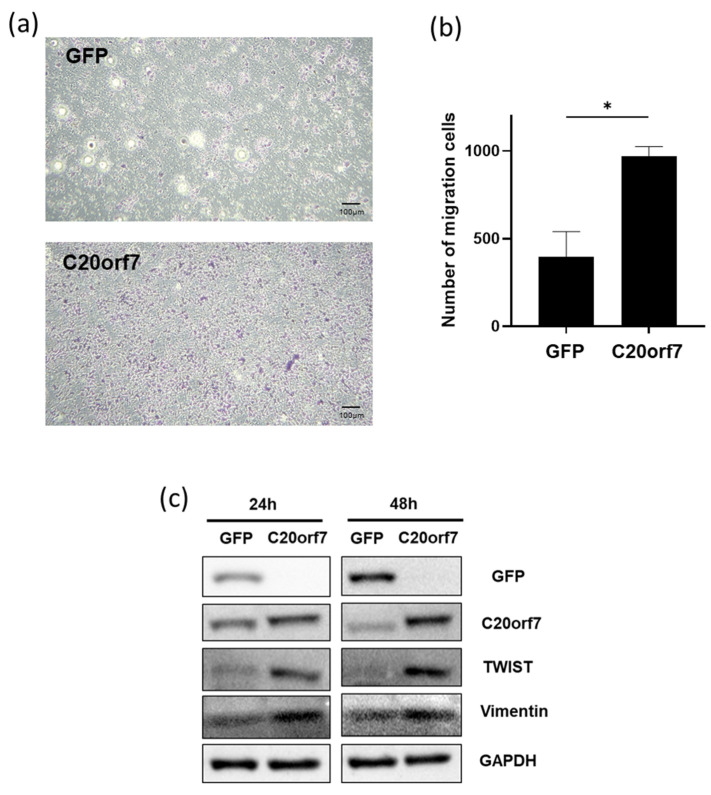
C20orf7 promotes the epithelial–mesenchymal transition (EMT) effect of colon cancer cells. (**a**) DLD1 cells were transfected with GFP or C20orf7 plasmids and then applied in Transwell migration assay; (**b**) the numbers of migrated cells were analyzed in the bar chart; (**c**) protein expression analysis by Western blot assay with control or C20orf7 overexpression samples at 24 h and 48 h time points. GAPDH was used as control. The results are presented as mean ± standard deviation (SD). * *p* < 0.05 was considered statistically significant. The scale bars indicate 100 µm.

Next, we further investigated whether C20orf7 could promote the vertical migration of cancer cells under the attraction of nutritional and macromolecular factors from FBS by Transwell migration assay. DLD1 colon cancer cells were transfected with GFP control plasmids or C20orf7-overexpressing plasmids in this experiment. The results showed that C20orf7-overexpressing cells resulted in significantly more migrated cells compared to the control (Figure 3a,b). As EMT plays a critical role in cancer metastasis, we examined whether the expression of C20orf7 regulates EMT markers. DLD1 cells were transfected with GFP control plasmids or C20orf7-overexpressing plasmids, and then harvested at 24h and 48h time-points for Western blot protein analysis. The results showed that the overexpression of C20orf7 indeed upregulated the levels of EMT progression markers, TWIST and Vimentin (Figure 3c). The results suggested that C20orf7 facilitates cancer migration, likely by regulating EMT progression in colon cancer.

### 3.4. C20orf7 Promotes Cell Proliferation and Chemotherapy Resistance in Colon Cancer Cells

**Figure 4:**
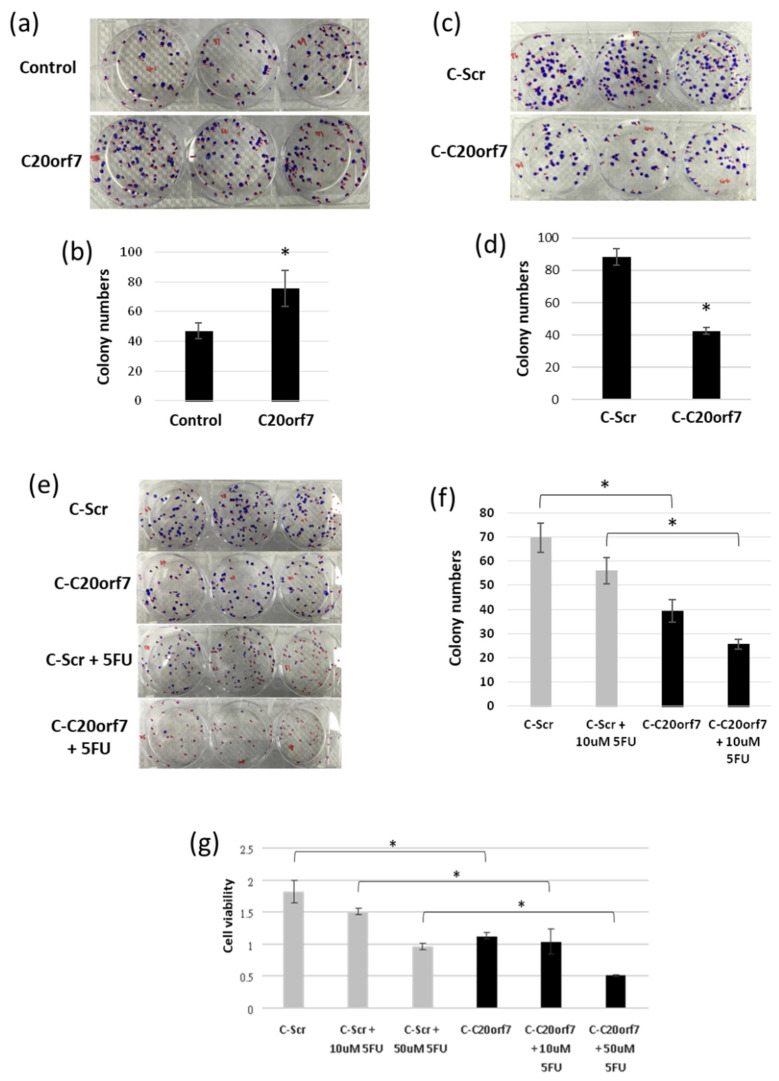
C20orf7 promotes cell proliferation and chemotherapy resistance in colon cancer cells. (**a**) Control or C20orf7 plasmids were overexpressed in cancer cells, and colony formation assay was performed; (**b**) the numbers of the colonies from the experiment in (**a**) were analyzed in the bar chart; (**c**) C-Scr and C-C20orf7 cells were seeded, and colony formation assay was performed; (**d**) the numbers of the colonies from the experiment in (**c**) were analyzed in the bar chart; (**e**) C-Scr and C-C20orf7 cells were treated with 5-fluorouracil (5FU) as indicated, and then applied in colony formation assay; (**f**) the numbers of the colonies from the experiment in (**e**) were analyzed; (**g**) C-Scr and C-C20orf7 cells were treated with 5FU as indicated, and then applied in MTS assay. The results are presented as mean ± standard deviation (SD). * *p* < 0.05 was considered statistically significant.

Next, we studied whether the expression of C20orf7 influences the proliferation of cancer cells by colony formation assay and MTS assay. The results showed that overexpression of C20orf7 transiently led to increased colony numbers, while the depletion of C20orf7 resulted in fewer colony numbers compared to control C-Scr cells (Figure 4a–d). The numbers of the colonies in these experiments were analyzed (Figure 4b,d). This suggested that C20orf7 promotes cancer cell proliferation. We then further examined whether the depletion of C20orf7 could sensitize the cancer inhibition effect of the common anti-cancer drug, 5FU. The results of the co-treatment experiment showed that the downregulation of C20orf7 resulted in fewer colonies compared to C-Scr, and 5FU treatment further depleted the colony numbers, suggesting that C20orf7 could sensitize the 5FU effect on suppressing cancer cells (Figure 4e,f). MTS assay was also performed with CRISPR cells and, consistently, the results indicated that the downregulation of C20orf7 suppresses cancer cell proliferation, and 5FU treatment further suppressed cell viability (Figure 4g). The above data indicate that C20orf7 can facilitate cell proliferation and chemotherapy resistance in colon cancer cells.

### 3.5. C20orf7 Contributes to Tumor Growth in the Xenograft Mice Model

**Figure 5:**
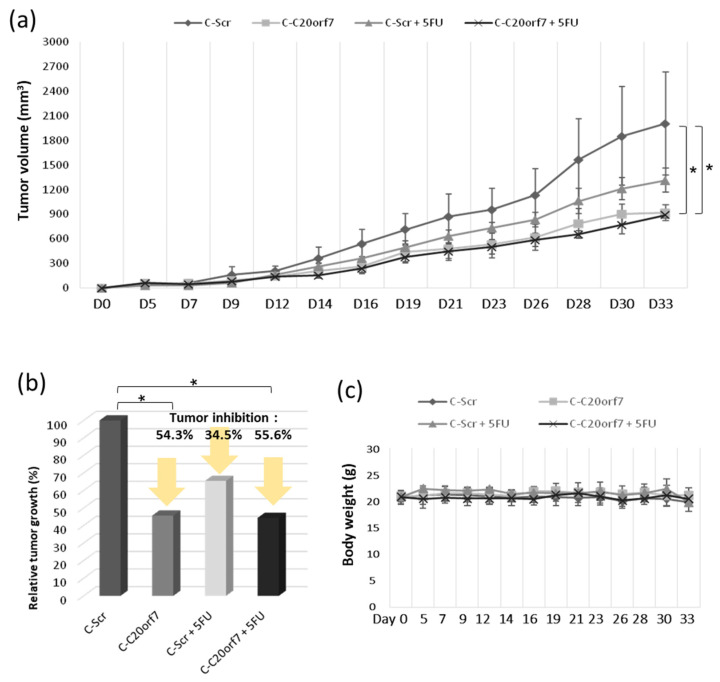
C20orf7 contributes to tumor growth in the xenograft mice model. C-Scr and C-C20orf7 cells were applied in the xenograft experiment. The 5FU was used as indicated and as described in the Materials and Methods. (**a**) The tumor growth of the mice in each group was measured during the experiment; (**b**) the tumor inhibition in different groups was analyzed; (**c**) the body weight of the nude mice in each group was monitored during the experiment. * *p* < 0.05 was considered statistically significant.

Since our results suggested that C20orf7 promotes the migration and proliferation of colon cancer cells, it was of interest to verify whether the expression of C20orf7 is critical to tumor growth in vivo. CRISPR cells were applied in the mice xenograft experiment with or without 5FU treatment as described in the Materials and Methods. The tumor growth was examined, and the average tumor size in each treatment ranged from large to small, in the following order: C-Scr > C-Scr plus 5FU > C-C20orf7 = C-C20orf7 plus 5FU (Figure 5a). Tumor inhibition was analyzed, and the results showed that, even though both C-C20orf7 and C-C20orf7 plus 5FU groups significantly suppressed tumor growth compared to C-Scr (Figure 5b), they were close to but did not reach the standard effective tumor inhibition definition from the NCI [31]. The body weight in each group remained stable during the experiment, indicating that none of the treatments resulted in strong toxicity reactions for these animals (Figure 5c). Hematologic toxicity, especially reductions in white blood cells, occurred in some human patients under 5FU administration [33,34,35]. Therefore, a hematological analysis was performed as a control for this experiment to monitor the blood cell counts, including neutrophils, lymphocytes, monocytes and eosinophils, within the animals. None of the experimental groups exhibited notable differences, or hematologic toxicity, compared to the untreated control group. The above data suggest that C20orf7 is a promising therapeutic target for colon cancer treatment.

## 4. Discussion

Here, we report the first evidence of the molecular regulation of C20orf7 in colon cancer tumorigenesis, and the potential to take C20orf7 as a therapeutic target for cancer treatment. We applied CRISPR technology to establish stable control cells and cells with downregulated C20orf7 expression and found that C20orf7 positively promotes the EMT-mediated cell migration of colon cancer. Moreover, the downregulation of C20orf7 could suppress the proliferation of cancer cells and sensitize the cancer inhibition potential of 5FU treatment. Our data showed the oncogenic properties of C20prf7 in colon cancer cells and in the xenograft mice model. The tumor inhibition difference in the mice experiment between control and treatment groups was statistically significant but did not reach the “effective tumor inhibition” criteria (T/C% ratio equal to or less than 42%) of NCI [31]. This suggests that the downregulation of C20orf7 might benefit clinical treatment as an adjuvant instead of a single treatment in the future. The detailed molecular underlining mechanisms await exploration. Whether and how C20orf7 is involved in the tumorigenesis of other neoplasms, or whether the depletion of C20orf7 can sensitize the efficiency of chemotherapy agents other than 5FU has yet to be investigated and verified.

Although targeting mitochondrial factors could be a cancer treatment strategy [12,17], as C20orf7 functions as a mitochondrial respiratory factor [18,19,20], how the downregulation of C20orf7 would impact other mitochondrial factors, as well as respiratory complex assembly, and thereby influence the homeostasis of mitochondrial function, has yet to be investigated and clarified. Previous studies suggested that mutations in C20orf7 could cause mitochondrial disease [18,21]. Moreover, in a neuroblastoma (NB) study, a novel protein p113 was found to mediate the upregulation of C20orf7, and that the higher expression of C20orf7 was associated with a lower probability of overall survival in NB patients [36]. The findings in NB are compatible with our study on colon cancer cells. Whether tumor cells harbor wild-type or mutated C20orf7, and the subsequent functional regulations in cancer, is intriguing. Furthermore, a study investigated the gene regulation network of Yes-associated protein 1 in cancers, which applied the Gene Ontology (GO) pathway enrichment and revealed that C20orf7 could be involved in the oxidation reduction process [37]. Further investigation of the relationship between C20orf7 and the oxidation reduction process could provide us with a better understanding of the regulation of the mitochondrial respiratory complex in oxidation regulation, and possibly increase our knowledge of whether and how this modulation may contribute to cancer formation or progression.

Previous studies also suggest that C20orf7 belongs to the SAM-dependent methyltransferase family, which means that it could methylate proteins within the mitochondria [18,21]. Since the above studies described the potential of targeting C20orf7 for cancer treatment, the development of small molecular inhibitors for C20orf7 suppression could be the next step. There have been various promising examples of the development of protein methyltransferase inhibitors for cancer therapy, such as EZH2 and PRMT5 inhibitors, and some have undergone clinical trials [38,39]. Whether the C20orf7 is wild-type or mutant could affect its methyltransferase activity, and an investigation of which proteins are C20orf7 targets would bring us a better understanding of the functional regulation of C20orf7 in biomedical sciences.

## 5. Conclusions

In this study, we identified a mitochondrial factor, C20orf7, with oncogenic properties, and investigated the role of C20orf7 in vitro and in vivo, to demonstrate that it could serve as a therapeutic target in colon cancer. This study and the previous findings have provided several lines of C20orf7 clues regarding the involvement of tumorigenesis, and further investigation would fill the gaps in the knowledge of the importance of mitochondria in cancer progression, and could also be necessary to verify C20orf7 as an applicable target for cancer treatment. Studies of whether the expression of C20orf7 is associated with clinical data could also help us to define if C20orf7 could be a biomarker for colon or other cancers.

## Data Availability

Not applicable.
